# Commentary: The Neural Bases of Emotion Regulation

**DOI:** 10.3389/fpsyg.2016.00476

**Published:** 2016-03-31

**Authors:** Timothy R. Rice

**Affiliations:** Psychiatry, Icahn School of Medicine at Mount SinaiNew York, NY, USA

**Keywords:** defense mechanisms, implicit emotion regulation, psychodynamic psychotherapy, psychoanalysis, child development

Amit Etkin, Christian Büchel, and James Gross make a valuable contribution in their article “The neural bases of emotion regulation” in the November 2015 issue of *Nature reviews: Neuroscience* (Etkin et al., 2015). Their review article concludes a process of gradual elevation of implicit emotion regulation to an equal footing with explicit emotion regulation. Whereas, explicit processes of emotion regulation refers to those that demand conscious, effortful application, implicit refers to those that proceed automatically and unconsciously. In this commentary, I suggest that the increased recognition and neural definition of implicit emotion regulation processes offers a unique opportunity for psychodynamic psychotherapy. The postulated equivalence of defense mechanisms with implicit emotion regulation (Rice and Hoffman, 2014) extends an opportunity to ground a key psychodynamic construct in defined neural correlates.

Etkin and colleagues' article is not the first time that the implicit emotion regulation construct has been identified. In a 2011 article Etkin's group introduced “two theoretical and empirical spheres that organize different areas of emotion regulation and label these as ‘explicit’ and ‘implicit”’ (Gyurak et al., 2011). The authors note that there was some precedent to this organization in the work of Bargh and Williams' conceptualizations of effortful and automatic processes (Bargh and Williams, 2007), a work preceded by Bargh's early interests in automaticity in social cognition (Bargh, 1994). The organization of Bargh and Williams' work parallels those of Susan Andersen and others, where the study of transference phenomenon and other unconscious processes developed a valuable scientific base to key psychodynamic principles (Berk and Andersen, 2000; Andersen and Przybylinski, 2012).

The explicit-implicit distinction offers a similar opportunity for the psychodynamic construct of defense mechanisms. Gross notes that past studies of emotion regulation include Freud's studies of how people unconsciously defend against anxiety-inducing impulses (Gross, 2013). Etkin and colleagues' valuation of implicit process emphasizes these unconscious processes as central to the field of neurobiological study of emotion regulation. The distinction between ventromedially-mediated implicit processes and more dorsolaterally-mediated explicit processes in the prefrontal cortex offers a means to observe these distinct unconscious processes. Because implicit emotion regulation is postulated to be equivalent to defense mechanisms (Rice and Hoffman, 2014), a key psychodynamic construct gains a neural signature and a valuable place in the study of the affective neurosciences. Establishing brain-based, neurobiological correlates to traditionally psychological constructs advances the goals of the Research Domain Criteria (RDoC) project (Insel et al., 2010) and furthers the rapprochement between psychodynamics and contemporary medicine.

Implicit regulation originates in the inhibitory action of the ventral prefrontal cortex (vPFC), which includes the orbitofrontal cortex (OFC), ventromedial PFC (vmPFC), and ventral anterior cingulate cortex (vACC; Etkin et al., 2013). Bidirectional inhibitory action from these centers modulates lower brain structures including the amygdala, ventral striatum, hypothalamus, and brainstem nuclei. These pathways are distinct from those in explicit emotion regulation that originate in areas including the dorsal anterior cingulate cortex (dACC) and the dorsolateral PFC (dPFC). The successful modulation of these limbic and visceromotor centers by these PFC regulators leads to decreased sympathetic arousal and increased parasympathetic or vagal tone (Lane et al., 2009). This results in a measurable neurochemical state of phenotypic calmness.

Regional differentials in synaptogenesis, myelination, and pruning create earlier maturity in ventral as opposed to dorsal areas (Fuster, 2002), leaving children and adolescents without explicit cognitive emotional controls (Casey et al., 2011). Normative development in the implicit ER system thus may be extremely important in children to promote self-regulation when the neurobiological substrates to employ explicit ER are lacking.

Operationalized defense analysis may progress implicit ER development. Contributions beyond Freud, particularly those made in the subspecialty of child and adolescent psychoanalysis, make the parallel between implicit emotion regulation and defense mechanisms clear. Sigmund Freud's daughter Anna significantly developed the defense mechanism construct (Freud, 1936). The shift from understanding defenses as protecting against theoretical, immeasurable drives to protecting against observable and measurable affective states occurred through the groundbreaking work of Bornstein (1945, 1949) and the subsequent generational line of child analysts to the present (Becker, 1974; Hoffman, 2007). This shift and its contemporary acceptance emphasize that children employ a range of defenses to help themselves tolerate unbearable, discomforting feelings. The parallels between implicit emotion regulation and defense mechanisms is quite clear.

To date, no author has offered a biological substrate to defense mechanisms, though broader efforts at defining the neural bases of the dynamic unconscious have been made (Berlin, 2011). This makes this contribution that is made possible by the scientific works of Etkin, Bargh, and Gross unique and valuable.

The relative isolation of the child psychoanalytic literature may have served to hinder awareness of the parallels between defense mechanisms and implicit emotion. This may have prevented the spread of established therapeutic interventions focused on defense mechanisms beyond psychoanalytic circles. The operationalization of defense analysis into a manualized, short-term psychotherapy termed Regulation Focused Psychotherapy for Children (RFP-C; Hoffman et al., 2016) is one means to interface this therapeutic intervention targeting implicit ER with contemporary models of health care provision. This approach maintains focus on the disruptive child's aggression as maladaptive defense mechanisms against sadness, loss, loneliness, and trauma. The approach helps the child to recognize that the feared feelings will not overwhelm him or her, and that alternative strategies are available. Through this approach children develop a wider range of adaptive defense mechanisms and employ them more flexibly, leading to health and developmental progression (Hoffman, 2007).

The observed parallels between defense mechanisms and the implicit emotion regulation system (Rice and Hoffman, 2014) enable us to hypothesize that this manualized procedure will strengthen implicit emotion regulation processes. Children who participate in the treatment will show improvements in validated measures of emotion regulation. Further elaboration of the implicit emotion regulation system and its neural correlates and of this psychodynamic treatment may one day enable measurement of change at the neural system level, through electroencephalogram or functional magnetic resonance imaging. The affective neurosciences offer rich opportunities for psychodynamic psychiatry, and Etkin and colleague's work offer a special one for defense analysis.

## Author contributions

The author confirms being the sole contributor of this work and approved it for publication.

### Conflict of interest statement

The author declares that the research was conducted in the absence of any commercial or financial relationships that could be construed as a potential conflict of interest.
